# Long-term Effect of Machine Learning–Triggered Behavioral Nudges on Serious Illness Conversations and End-of-Life Outcomes Among Patients With Cancer: A Randomized Clinical Trial

**DOI:** 10.1001/jamaoncol.2022.6303

**Published:** 2023-01-12

**Authors:** Christopher R. Manz, Yichen Zhang, Kan Chen, Qi Long, Dylan S. Small, Chalanda N. Evans, Corey Chivers, Susan H. Regli, C. William Hanson, Justin E. Bekelman, Jennifer Braun, Charles A. L. Rareshide, Nina O’Connor, Pallavi Kumar, Lynn M. Schuchter, Lawrence N. Shulman, Mitesh S. Patel, Ravi B. Parikh

**Affiliations:** 1 Department of Medical Oncology, Dana-Farber Cancer Institute, Boston, Massachusetts; 2 Harvard Medical School, Boston, Massachusetts; 3 Division of Health Policy, Perelman School of Medicine, University of Pennsylvania, Philadelphia; 4 Department of Biostatistics, Epidemiology, and Informatics, Perelman School of Medicine, University of Pennsylvania, Philadelphia; 5 Wharton School of the University of Pennsylvania, Philadelphia; 6 Department of Medicine, Perelman School of Medicine, University of Pennsylvania, Philadelphia; 7 Penn Medicine, Philadelphia, Pennsylvania; 8 Penn Center for Cancer Care Innovation, Abramson Cancer Center, University of Pennsylvania, Philadelphia; 9 Corporal Michael J. Crescenz Veterans Affairs Medical Center, Philadelphia, Pennsylvania; 10 Ascension Health, St Louis, Missouri

## Abstract

**Importance:**

Serious illness conversations (SICs) between oncology clinicians and patients are associated with improved quality of life and may reduce aggressive end-of-life care. However, most patients with cancer die without a documented SIC.

**Objective:**

To test the impact of behavioral nudges to clinicians to prompt SICs on the SIC rate and end-of-life outcomes among patients at high risk of death within 180 days (high-risk patients) as identified by a machine learning algorithm.

**Design, Setting, and Participants:**

This prespecified 40-week analysis of a stepped-wedge randomized clinical trial conducted between June 17, 2019, and April 20, 2020 (including 16 weeks of intervention rollout and 24 weeks of follow-up), included 20 506 patients with cancer representing 41 021 encounters at 9 tertiary or community-based medical oncology clinics in a large academic health system. The current analyses were conducted from June 1, 2021, to May 31, 2022.

**Intervention:**

High-risk patients were identified using a validated electronic health record machine learning algorithm to predict 6-month mortality. The intervention consisted of (1) weekly emails to clinicians comparing their SIC rates for all patients against peers’ rates, (2) weekly lists of high-risk patients, and (3) opt-out text messages to prompt SICs before encounters with high-risk patients.

**Main Outcomes and Measures:**

The primary outcome was SIC rates for all and high-risk patient encounters; secondary end-of-life outcomes among decedents included inpatient death, hospice enrollment and length of stay, and intensive care unit admission and systemic therapy close to death. Intention-to-treat analyses were adjusted for clinic and wedge fixed effects and clustered at the oncologist level.

**Results:**

The study included 20 506 patients (mean [SD] age, 60.0 [14.0] years) and 41 021 patient encounters: 22 259 (54%) encounters with female patients, 28 907 (70.5%) with non-Hispanic White patients, and 5520 (13.5%) with high-risk patients; 1417 patients (6.9%) died by the end of follow-up. There were no meaningful differences in demographic characteristics in the control and intervention periods. Among high-risk patient encounters, the unadjusted SIC rates were 3.4% (59 of 1754 encounters) in the control period and 13.5% (510 of 3765 encounters) in the intervention period. In adjusted analyses, the intervention was associated with increased SICs for all patients (adjusted odds ratio, 2.09 [95% CI, 1.53-2.87]; *P* < .001) and decreased end-of-life systemic therapy (7.5% [72 of 957 patients] vs 10.4% [24 of 231 patients]; adjusted odds ratio, 0.25 [95% CI, 0.11-0.57]; *P* = .001) relative to controls, but there was no effect on hospice enrollment or length of stay, inpatient death, or end-of-life ICU use.

**Conclusions and Relevance:**

In this randomized clinical trial, a machine learning–based behavioral intervention and behavioral nudges to clinicans led to an increase in SICs and reduction in end-of-life systemic therapy but no changes in other end-of-life outcomes among outpatients with cancer. These results suggest that machine learning and behavioral nudges can lead to long-lasting improvements in cancer care delivery.

**Trial Registration:**

ClinicalTrials.gov Identifier: NCT03984773

## Key Points

**Question:**

Does an intervention consisting of machine learning mortality predictions and behavioral nudges to oncology clinicians have an impact on serious illness conversations (SICs) and end-of-life outcomes among patients with cancer?

**Findings:**

In this randomized clinical trial that included 20 506 patients with cancer (for a total of 41 021 encounters), the intervention led to a significant increase in SICs, from 3.4% to 13.5%, among encounters with patients at high risk of death while decreasing rates of end-of-life systemic therapy from 10.4% to 7.5%.

**Meaning:**

These results suggest that a machine learning–based behavioral intervention can lead to an increase in SICs and reduction in end-of-life systemic therapy among outpatients with cancer.

## Teaser

This randomized clinical trial assesses the impact of behavioral nudges to clinicians on the rate of serious illness conversations and end-of-life outcomes among patients at high risk of death.

## Introduction

Serious illness conversations (SICs) between oncology clinicians and patients with cancer focus on patients’ understanding of and priorities around their illness.^1^ These conversations are appropriate at any stage of cancer, improve mood and quality of life, and may reduce end-of-life systemic therapy use.^2,3^ However, most patients die without an SIC.^4^ Clinician factors, including inaccurate prognostication and perceptions that SICs are appropriate only close to death, contribute to this dearth of SICs.^5^ To address these factors, we conducted a stepped-wedge, cluster-randomized clinical trial that evaluated behavioral nudges to clinicians to prompt SICs among patients at high risk for death within 180 days (high-risk patients) identified by a machine learning algorithm.^6^ A prior analysis after 16 weeks showed significant increases in SICs among all and high-risk patients. Here we report the intervention impact on prespecified 40-week SIC and end-of-life outcomes.

## Methods

**Figure 1.**
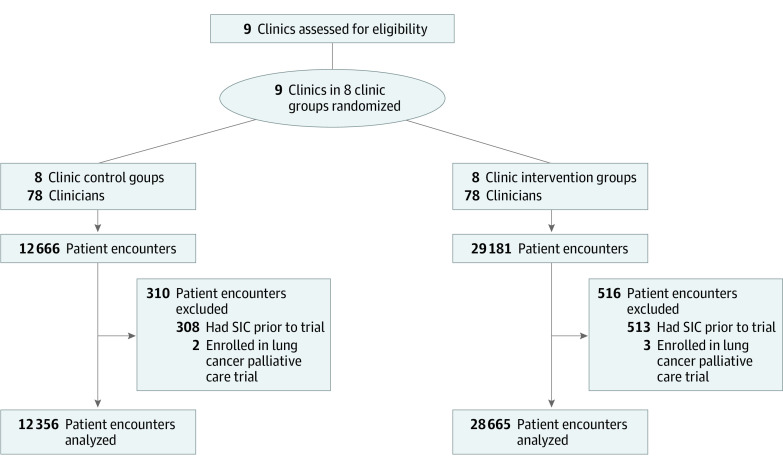
Randomization Flowchart SIC indicates serious illness conversation.

The 16-week results of this trial were previously published.^6^ The trial protocol was approved by the University of Pennsylvania institutional review board (Supplement 1), which granted a waiver of informed consent owing to minimal risk. Patients with encounters at 1 of 9 tertiary and community-based medical oncology clinics between June 17, 2019, and April 20, 2020, without a prior documented SIC were eligible. The current analyses were conducted from June 1, 2021, to May 31, 2022. Details of randomization are shown in Figure 1. We identified high-risk patients using a prospectively validated machine learning algorithm predicting 6-month mortality; patients with a 10% or higher absolute mortality risk were deemed high risk.^6,7^ The intervention consisted of (1) weekly emails to clinicians comparing their SIC rates for all patients against peers’ rates, (2) weekly lists of 6 or more forthcoming encounters with high-risk patients, and (3) opt-out reminder texts to clinicians on the morning of encounters with high-risk patients. While emails reported SICs for all patients, opt-out text messages only applied to encounters with high-risk patients. After a 4-week baseline wedge in which all groups remained in usual care (control), groups were randomly assigned to the intervention in 4-week wedges until all clinics received the intervention by the fifth wedge (week 17) and were followed up through week 40.

Primary outcomes at 40 weeks (after 16 weeks of rollout and 24 weeks of follow-up) included SIC rates for all, high-risk, and non–high-risk patients, along with subgroup analyses stratified by age, self-reported race and ethnicity, sex, insurance status, and clinic type. A specific type of note indicated SICs in the advance care planning module of the electronic medical record. End-of-life outcomes included inpatient death, intensive care unit use within 30 days of death, systemic therapy (chemotherapy or checkpoint inhibitor therapy) within 14 days of death, hospice enrollment prior to death, and hospice length of stay.^8,9^ End-of-life outcomes were derived from an institutional cancer registry and were available for 1188 patients with 3 or more oncology encounters in the year prior to death; hospice outcomes were available for 856 patients eligible for a health system–affiliated hospice. Decedents were assigned to control vs intervention group based on intervention status on the date of the last clinic encounter. Outcomes were analyzed at either the patient-encounter (ie, SIC) or patient (end-of-life) level using generalized estimating equations with a logit link (binary outcomes) or gamma link (continuous outcomes) and an independent correlation structure using oncologist as the clustering unit to account for between-clinician variation, with clinic-group and wedge-period fixed effects. Heterogeneity of intervention effects was evaluated using (subgroup × intervention) interaction terms. Analyses were performed using R statistical software, version 4.2.1 (R Project for Statistical Computing). A 2-tailed *P* < .05 was considered statistically significant.

## Results

**Figure 2.**
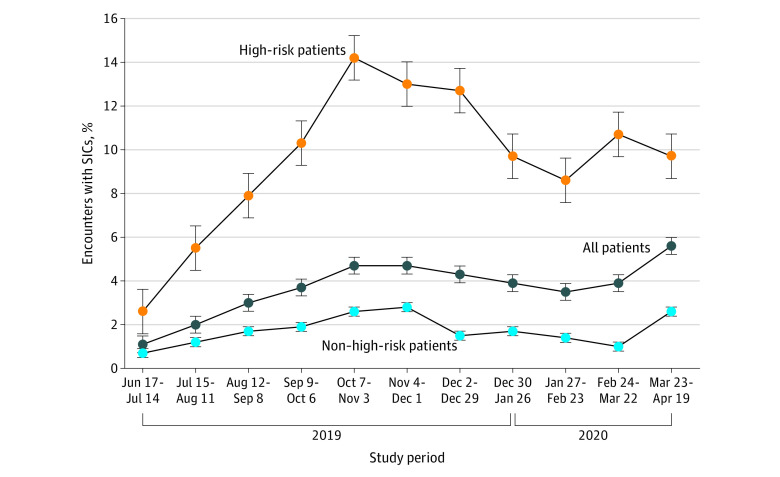
Rates of Serious Illness Conversations (SICs) Over the Study Period High-risk patients were defined as those at high risk of death within 180 days as identified by a machine learning algorithm. The intervention rollout began at the 16-week mark. Filled circles represent the percent of monthly encounters with recorded SIC completion. Whiskers indicate SEs.

The study included 20 506 patients (mean [SD] age, 60.0 [14.0] years) and 41 021 patient encounters: 22 259 (54%) encounters with female patients, 8175 (19.9%) with non-Hispanic Black patients, 28 907 (70.5%) with non-Hispanic White patients, and 3939 (9.6%) with patients categorized as other (including American Indian, Asian, East Indian, Hispanic Black, Hispanic White, and Pacific Islander); 5520 encounters (13.5%) were with high-risk patients (eTable 1 in Supplement 2). There were no meaningful baseline differences between control and intervention periods. For the overall study population, SIC rates increased during the initial rollout and plateaued at a higher baseline in the follow-up period (Figure 2). Among all patient encounters, the unadjusted SIC rates were 1.3% (158 of 12 356 encounters) and 4.4% (1262 of 28 665 encounters) in the control and intervention periods, respectively (Table). Among encounters with high-risk patients, the unadjusted SIC rate was 3.4% (59 of 1754 encounters) and 13.5% (510 of 3765 encounters) in the control and intervention periods, respectively. Among encounters with non–high-risk patients, the unadjusted SIC rates were 0.9% (99 of 10 602 encounters) and 3.0% (752 of 24 899 encounters) in the control and intervention periods, respectively. The intervention was associated with significant increases in SICs among all patient encounters (adjusted odds ratio [aOR], 2.09 [95% CI, 1.53-2.87]; *P* < .001), those with high-risk patients (aOR, 2.62 [95% CI, 1.84-3.72]; *P* < .001), and those with non–high-risk patients (aOR, 2.07 [95% CI, 1.52-2.82]; *P* < .001). The intervention effect was significant across all subgroups (eTable 2 in Supplement 2); interaction models revealed a greater impact on SIC rates among Medicaid beneficiaries (5.2% of encounters in the intervention group vs 0.9% in the control group; aOR, 2.19 [95% CI, 1.10-4.37]; *P* = .03 for interaction). No other significant interaction effects were found. There were 419 SICs (29.5% of all SICs) among patients who died; the unadjusted SIC rates were 19.5% (42 of 215 patients) and 31.4% (377 of 1202 patients) in the control and intervention periods, respectively (aOR, 2.44 [95% CI, 1.35-4.42]; *P* < .001).

**Table.** Adjusted Changes in Serious Illness Conversations and End-of-Life Outcomes

	No./total No. (%) of encounters		Odds of an SIC, aOR (95% CI)^a^	*P* value
	Control period	Intervention period		
**SICs**				
All patient encounters	158/12 356 (1.3)	1262/28 665 (4.4)	2.09 (1.53-2.87)	<.001
Encounters with high-risk patients^b^	59/1754 (3.4)	510/3765 (13.5)	2.62 (1.84-3.72)	<.001
Encounters with non–high-risk patients	99/10 602 (0.9)	752/24 899 (3.0)	2.07 (1.52-2.82)	<.001
Deceased patients	42/215 (19.5)	377/1202 (31.4)	2.44 (1.35-4.42)	<.001
**End-of-life outcomes**				
All patients				
Inpatient death^c^	27/231 (11.7)	114/957 (11.9)	1.72 (0.7-4.24)	.24
ICU admission within 30 d of death^c^	39/231 (16.9)	150/957 (15.7)	1.04 (0.54-1.98)	.92
Enrolled in hospice^c^	93/156 (59.6)	424/700 (60.6)	1.42 (0.78-2.59)	.25
Hospice length of stay, mean (SD), d^d^	23.90 (40.77)	25.53 (37.01)	1.26 (0.67-2.37)	.47
Systemic therapy within 14 d of death^c^	24/231 (10.4)	72/957 (7.5)	0.25 (0.11-0.57)	.001
High-risk patients				
Inpatient death	22/181 (12.2)	83/696 (11.9)	1.8 (0.63-5.13)	.27
ICU admission within 30 d of death	28/181 (15.5)	120/696 (17.2)	1.12 (0.51-2.45)	.77
Enrolled in hospice^c^	79/124 (63.7)	340/525 (64.8)	1.53 (0.72-3.25)	.27
Hospice length of stay, mean (SD), d^d^	26.33 (43.53)	25.03 (36.65)	1.24 (0.64-2.41)	.53
Systemic therapy within 14 d of death^c^	15/181 (8.3)	50/696 (7.2)	0.37 (0.13-1.04)	.06
Non–high-risk patients				
Inpatient death	5/50 (10)	31/261 (11.9)	1.03 (0.62-1.69)	.92
ICU admission within 30 d of death	11/50 (22)	30/261 (11.5)	0.96 (0.63-1.47)	.87
Enrolled in hospice^c^	14/32 (43.8)	84/175 (48)	1.31 (0.96-1.79)	.08
Hospice length of stay, mean (SD), d^d^	25.03 (33.46)	26.39 (35.46)	0.68 (0.18-2.56)	.57
Systemic therapy within 14 d of death	9/70 (12.90)	22/347 (6.30)	0.84 (0.44-1.61)	.61

Abbreviations: aOR, adjusted odds ratio; ICU, intensive care unit; SIC, serious illness conversation.

^a^ Adjusted for intervention, clinic group, and wedge, and clustered at the oncologist level.

^b^ High-risk patients were defined as having a 10% or greater risk of death within 180 days, as identified by a prospectively validated machine learning algorithm.

^c^ End-of-life inpatient and systemic therapy data available for 1188 patients.

^d^ Hospice outcomes available for 856 patients who were eligible for health system–affiliated hospice.

There were 1417 deaths by the end of follow-up (6.9% of patients), 795 (56.1%) of which were in high-risk patients (mortality rate among high-risk patients, 35.2% [795 of 2261 patients]; rate among non–high-risk patients, 3.4% [622 of 18 245 patients]). Intervention patients were less likely than control patients to receive systemic therapy at the end of life (all decedents: 7.5% [72 of 957 patients] vs 10.4% [24 of 231 patients]; aOR, 0.25 [95% CI, 0.11-0.57]; *P* = .001; high-risk decedents: 7.2% [50 of 696 patients] vs 8.3% [15 of 181 patients]; aOR, 0.37 [95% CI, 0.13-1.04]; *P* = .06) (Table). There were no differences between control and intervention groups in other end-of-life outcomes.

## Discussion

In this randomized clinical trial, a machine learning–based intervention increased SICs and decreased end-of-life systemic therapy among patients with cancer. Our findings suggest that machine learning–based interventions may lead to long-term reduction in low-value cancer care and offer several lessons.^10^

First, our intervention led to a sustained increase in SICs, and rates remained nearly 4 times above baseline after 6 months. A prior effort to improve serious illness communication has found inconsistent long-term results.^2^ Our intervention’s sustained effect on SICs likely stemmed from the integration of proven behavioral interventions, including peer comparisons,^11^ performance reports,^12^ and opt-out reminders.^13^ Of note, our intervention increased SICs even among patients not flagged by the algorithm (ie, a spillover).

Second, our subgroup analyses suggest that intervention effects were as strong or stronger among Medicaid beneficiaries, who have historically lower SIC rates.^14^ Our algorithm-based intervention may have had a larger impact on disadvantaged groups by addressing clinician-level biases that contributed to lower baseline SIC rates.

Third, our intervention decreased end-of-life systemic therapy. This impact stands in contrast to a prior trial,^2^ which found no impact of SIC education alone on end-of-life outcomes. Practices participating in performance-based payment models may find that machine learning–based interventions facilitate higher-quality end-of-life care.

### Limitations

This study has limitations. First, this single–health system trial may not be generalizable to other systems. Second, our intervention’s impact on SIC and end-of-life outcomes may have been limited because it nudged time-constrained oncologists. Future machine learning–based nudges could involve specialty palliative care referral and nonclinician staff. Third, there are no established standards defining a clinically meaningful impact on end-of-life systemic therapy. However, the effect estimate for end-of-life systemic therapy compares similarly to a prior randomized evaluation of early palliative care.^15^ Finally, SICs have improved mental health, quality of life, and emotional distress in prior work,^1,2^ but our study did not measure these outcomes. Future prospective SIC studies should prioritize patient-reported outcome measurement.

## Conclusions

In this randomized clinical trial, a machine learning–based behavioral intervention led to a sustained increase in SICs and reduction in end-of-life systemic therapy among outpatients with cancer, suggesting that machine learning–based interventions can lead to long-lasting improvements in cancer care delivery.

## Notes

Supplement 1.
Trial Protocol

Supplement 2. **eTable 1. **Characteristics of Patients With Clinician Encounters **eTable 2.** Subgroup Analyses

Supplement 3.
Data Sharing Statement
