# Supplementary material for: Long-term Effect of Machine Learning–Triggered Behavioral Nudges on Serious Illness Conversations and End-of-Life Outcomes Among Patients With Cancer: A Randomized Clinical Trial
Source: JAMA Oncol. 2023 Jan 12;9(3):414–8. doi: 10.1001/jamaoncol.2022.6303 (PMC9857721; doi:10.1001/jamaoncol.2022.6303)
Supplement: Supplement 1. — Trial Protocol [file jamaoncol-e226303-s001.pdf]

A Stepped-Wedge Cluster Randomized Trial Using Machine-Generated Mortality Estimates and Behavioral Nudges to Promote Advance Care Planning Discussion among Cancer Patients

Study Protocol

June 2019

Outline

1. Abstract
2. Overall objectives
3. Aims
    - 3.1 Primary outcome
    - 3.2 Secondary outcomes
4. Background
5. Study design
    - 5.1 Design
    - 5.2 Study duration
    - 5.3 Target population
    - 5.4 Accrual
    - 5.5 Key inclusion criteria
    - 5.6 Key exclusion criteria
6. Subject recruitment
7. Subject compensation
8. Study procedures
    - 8.1 Consent
    - 8.2 Procedures
9. Analysis plan
10. Investigators
11. Human research protection
    - 11.1 Data confidentiality
    - 11.2 Subject confidentiality
    - 11.3 Subject privacy

11.4 Data disclosure

11.5 Data safety and monitoring

11.6 Risk/benefit

    11.6.1 Potential study risks

    11.6.2 Potential study benefits

    11.6.3 Risk/benefit assessment

1. Abstract

Patients with cancer often undergo costly therapy and acute care utilization that is discordant with their wishes, particularly at the end of life. Early serious illness conversations (SIC) improve goal-concordant care, and accurate prognostication is critical to inform the timing and content of these discussions. In this project, we will evaluate a health system initiative that uses a machine learning algorithm to predict patients with a higher risk of short-term mortality and then prompts oncologists to SICs with these patients. In partnership with the health system, this will be conducted as a cluster-randomized trial to evaluate its effect.

2. Overall objectives

The objective of the study is to evaluate the effect of a health system initiative using machine learning algorithms and behavioral nudges to prompt oncologists to have serious illness conversations with patients at high-risk of short-term mortality.

3. Aims

3.1 Primary outcome

The primary outcome is change in the proportion of patients that have an outpatient oncology visit with documentation of a serious illness conversation (SIC).

3.2 Secondary outcome

The secondary outcomes are: 1) the change in the proportion of patients who have an outpatient oncology visit and are identified as high-risk by the machine learning algorithm with documentation of a SIC; 2) the change in the proportion of patients that have an outpatient oncology visit with documentation of advanced care planning (ACP).

3.3 Exploratory outcomes

- Acute care utilization metrics, including Oncology Evaluation Center, ED, Inpatient and ICU admissions.
- Healthcare utilization in the last 30 days of life in Penn Medicine facilities: acute care utilization as above, plus receipt of chemotherapy in the last 30 days.

4. Background

Patients with cancer often undergo costly therapy and acute care utilization that is discordant with their wishes, particularly at the end of life.[1–5] Early serious illness conversations (SICs) to determine a patient's goals and values for therapy can increase goal-concordant care.[6, 7] Nevertheless most patients with advanced cancer die without a documented SIC, including the vast majority of UPHS oncology patients in 2018.[8, 9] <u>A key reason for this gap may be that oncologists routinely overestimate life expectancy of patients with advanced cancer, delaying SICs until late in the disease course and resulting in aggressive care near the end of life.</u>[10, 11]

Existing prognostic aids in oncology are rarely used because they do not apply to most cancers[12, 13], do not identify most patients who will die within 1 year[14], and require time-consuming data input[15]. Electronic health record (EHR)-based predictive algorithms can improve clinician decision-making in acute care settings[16–18], but it is unclear whether such algorithms can guide clinicians to perform SICs. As oncologists strive to assess patients' goals earlier in the disease course, accurate prognostication is critical to inform the timing and content of these discussions.

--References--

1. Emanuel EJ, Young-Xu Y, Levinsky NG, et al: Chemotherapy use among Medicare beneficiaries at the end of life. Ann Intern Med 138:639–643, 2003

2. Earle CC, Neville BA, Landrum MB, et al: Trends in the aggressiveness of cancer care near the end of life. J Clin Oncol 22:315–321, 2004

3. Earle CC, Landrum MB, Souza JM, et al: Aggressiveness of cancer care near the end of life: is it a quality-of-care issue? J Clin Oncol 26:3860–3866, 2008

4. Chastek B, Harley C, Kallich J, et al: Health Care Costs for Patients With Cancer at the End of Life. JOP 8:75s-80s, 2012

5. Wen F-H, Chen J-S, Su P-J, et al: Terminally Ill Cancer Patients' Concordance Between Preferred Life-Sustaining Treatment States in Their Last Six Months of Life and Received Life-Sustaining Treatment States in Their Last Month: An Observational Study. J Pain Symptom Manage 56:509-518.e3, 2018

6. Wright AA, Zhang B, Ray A, et al: Associations between end-of-life discussions, patient mental health, medical care near death, and caregiver bereavement adjustment. JAMA 300:1665–1673, 2008

7. Brinkman-Stoppelenburg A, Rietjens JAC, van der Heide A: The effects of advance care planning on end-of-life care: a systematic review. Palliat Med 28:1000–1025, 2014

8. National Quality Forum: Palliative and end-of-life care 2015-2016. [Internet]. Washington, DC, National Quality Forum, 2016[cited 2018 Aug 12] Available from: http://www.qualityforum.org/Projects/n-r/Palliative_and_End-of-Life_Care_Project_2015-2016/Draft_Report_for_Comment.aspx

9. Schubart JR, Levi BH, Bain MM, et al: Advance Care Planning Among Patients With Advanced Cancer. JOP JOP.18.00044, 2018

10. Christakis NA, Smith JL, Parkes CM, et al: Extent and determinants of error in doctors' prognoses in terminally ill patients: prospective cohort studyCommentary: Why do doctors overestimate?Commentary: Prognoses should be based on proved indices not intuition. BMJ 320:469–473, 2000

11. Sborov K, Giaretta S, Koong A, et al: Impact of Accuracy of Survival Predictions on Quality of End-of-Life Care Among Patients With Metastatic Cancer Who Receive Radiation Therapy. JOP JOP.18.00516, 2019

12. Fong Y, Evans J, Brook D, et al: The Nottingham Prognostic Index: five- and ten-year data for all-cause Survival within a Screened Population. Ann R Coll Surg Engl 97:137–139, 2015

13. Alexander M, Wolfe R, Ball D, et al: Lung cancer prognostic index: a risk score to predict overall survival after the diagnosis of non-small-cell lung cancer. Br J Cancer 117:744–751, 2017

14. Lakin JR, Robinson MG, Bernacki RE, et al: Estimating 1-Year Mortality for High-Risk Primary Care Patients Using the "Surprise" Question. JAMA Intern Med 176:1863–1865, 2016

15. Morita T, Tsunoda J, Inoue S, et al: The Palliative Prognostic Index: a scoring system for survival prediction of terminally ill cancer patients. Support Care Cancer 7:128–133, 1999

16. Parikh RB, Kakad M, Bates DW: Integrating Predictive Analytics Into High-Value Care: The Dawn of Precision Delivery. JAMA 315:651–652, 2016

17. Amarasingham R, Audet A-MJ, Bates DW, et al: Consensus Statement on Electronic Health Predictive Analytics: A Guiding Framework to Address Challenges [Internet]. EGEMS (Wash DC) 4, 2016[cited 2019 Jan 11] Available from: https://www.ncbi.nlm.nih.gov/pmc/articles/PMC4837887/
18. Bates DW, Saria S, Ohno-Machado L, et al: Big Data In Health Care: Using Analytics To Identify And Manage High-Risk And High-Cost Patients. Health Affairs 33:1123–1131, 2014

5. Study design

5.1 Design

This study will use a stepped-wedge cluster randomized trial to evaluate a health system initiative. Oncology practices will be randomly assigned in sequential four-week blocks to receive the email prompt intervention, in which individual oncologists will receive an automated weekly email detailing 1) how many serious illness conversations they have had, 2) how their number of serious illness conversations compares to peer oncology providers across UPHS, and 3) a weekly roster of their upcoming patients at high risk of short-term mortality as determined by our mortality prediction algorithm (see below), viewable on a HIPAA-compliant secure web interface. Clinicians will receive a HIPAA compliant text message on the morning of the appointment reminding them to consider a serious illness conversation with patients on the list. Providers may opt out of this reminder on the web interface containing the weekly patient roster of high risk patients. Prior to receiving the intervention, practices will receive current standard communications regarding serious illness performance until they are randomized to the intervention. Practices will be cluster-randomized to the intervention over a 16-week period, after which all practice physicians will receive the email intervention.

Based on clinician and health system feedback, the intervention will be modified to remove the peer comparison message. This will occur 12 weeks into the follow-up period.

Following the intervention, brief REDCap questionnaires will be sent to all clinicians who participated in the trial to explore perceptions of the study intervention. This survey should take no longer than five minutes for clinicians to complete and will assess their feedback on the overall intervention in addition to specific components including automated identification of patients, receiving text and email notifications, and identifying the appropriate patients. The survey will also collect basic demographic information (age, gender, practice site, and comfort with SICs).

Additionally, we plan to contact clinicians who participated in the intervention and invite them to share their experiences with us via a semi-structured interview. The interview guide (see **Conversation Connect Initiative Clinician Interview Guide**) was developed with feedback from the Mixed Methods Research Lab (MMRL), an organization that specializes in qualitative research methods, analysis, and human subject protections. The interview guide includes questions focused on clinicians' comfort level with SICs prior to the intervention, specific components (emails and text messages), and additional strategies that could be implemented to

improve rates of SICs with patients. In summary, we will use semi-structured interviews to elicit clinicians' perspectives on the Conversation Connect intervention beyond the brief REDCap surveys. Additionally, clinicians will be asked basic demographic questions regarding their age, race, gender, practice setting, and number of years in practice. We will pilot this guide with 3-5 oncology clinicians and anticipate it will take approximately 20 minutes to complete. The guide may be revised to better clarify questions or adjust timing during the initial first five subject interviews. Clinicians will be interviewed at a mutually convenient time by phone. Each interview will be audio-recorded with permission from the provider and subsequently transcribed for analysis. All research personnel will have completed human subject protection modules prior to initiating the study. The MMRL will conduct all of the interviews. The MMRL will review transcripts of the interviews and iteratively develop the code book and analyze the content of the interviews.

Additionally, we will plan on studying the impact of the COVID-19 pandemic on the output and accuracy of our predictive model, and identify subpopulations for whom utilization declines during COVID have led to the most decrements in the predictive accuracy of the Conversation Connect predictive model. We will specifically assess heterogeneity by racial/ethnic groups (White, Black, Hispanic, Asian, and Native American), insurance status (Medicare, Medicaid, Commercial), low-income zip code, and area-level socioeconomic metrics (e.g. area-level income level), in addition to by cancer type. We will also assess the impact of COVID-related changes in predictive model output on the rate of Serious Illness Conversations documented as part of the trial.

5.2 Study duration

The study is expected to begin in June 2019 and take 10 months (16 weeks for intervention + 24 weeks followup) to complete. The REDCap questionnaires are expected to be completed by March 2021. The semi-structured interviews will take place from December 2020 to June 2021. Our analysis of COVID-related impacts will take place from February 2021 to July 2021.

5.3 Target population

Medical oncology clinicians (physicians, nurse practitioners and physician assistants) and their patients at the University of Pennsylvania Health System practicing at one of two hematology/oncology practices: The Perelman Center for Advanced Medicine (PCAM) and Pennsylvania Hospital (PAH).

5.4 Accrual

Patients will accrue to the trial as their clinical practice receives the email intervention. Eight University of Pennsylvania oncology practices will be randomly assigned to one of four start dates separated by four weeks, resulting in four pairs of clinics starting the intervention two clinics at a time every four weeks over sixteen weeks. When a clinic reaches the assigned start

date for the intervention arm, the clinicians will begin to receive the weekly email intervention and text reminders. Based on previous studies and assuming a baseline SIC rate of 0.65 SICs per provider per 4-weeks, we believe we will have over 80% power to detect a 60% increase in SIC rates per provider per 4-weeks.

5.5 Key inclusion criteria

Oncologists must meet the following criteria to be eligible for the study:

1) Care for adults with cancer at the following oncology clinics at the University of Pennsylvania Health System:

1. Perelman Center for Advanced Medicine:
    - Breast Oncology
    - Gastrointestinal Oncology
    - Genitourinary Oncology
    - Lymphoma
    - Melanoma and Central Nervous System Oncology (grouped together due to low number of providers)
    - Myeloma
    - Thoracic / Head and Neck Oncology (one group, not a combination of subspecialties)
2. Pennsylvania Hospital Oncology

5.6 Key exclusion criteria

- Providers at these clinics who care for only patients with benign hematologic disorders or who only see genetics consults will be excluded and not receive any emails.
- Providers who see less than 12 high-risk patients in either the pre- or post-intervention periods
- Visits for patients with lung cancer who are enrolled in an ongoing palliative care clinical trial that may lead to more SICs.
- Patient visits that are for oncology genetics consults (such patients may still be included if they see their primary oncologist during the trial)
- Providers who have not undergone Serious Illness Conversation Program training

6. Subject recruitment

Information on oncology practices and their clinicians at the University of Pennsylvania Health system will be identified by department leadership. High-risk patients will be identified by applying our mortality prediction algorithm (which uses electronic health record data from Clarity, an EPIC reporting database) to weekly oncology clinic schedules.

7. Subject compensation

No compensation will be offered in the intervention or REDCap survey. We will offer clinicians who participate in the semi-structured interviews a compensation of a $30 gift card.

8. Study procedures

8.1 Consent

A waiver of informed consent is requested. This is a health system initiative that will be implemented. The study is to evaluate that initiative. Therefore, physicians and their patients will not be consented as this is the standard of practice per the health system initiative. Without a waiver of the consent, the initiative would still be implemented by the health system, but the study would be infeasible. There are several additional reasons why we feel a waiver of consent should be granted. First, it is not feasible to consent every physician and as mentioned this initiative would occur with or without the study of it. Second, if members of the control group were consented, this alone could change their behavior. This could potentially disrupt the design of the study and making interpretation of the findings challenging. Third, physicians are not being forced to have serious illness conversations for their patients. Instead, they are being reminded of their patients at high-risk of mortality and receiving an email prompt regarding the number of serious illness conversations that they have had, , with opt-out text message reminders on the day of the appointment. This is no different than standard of care in which a physician would review the same information and decide to have a serious illness conversation. The initiative is simply a reminder for the physician and makes their standard of care process easier to conduct. Finally, as part of a previous quality improvement initiative, we previously interviewed 40 patients after a serious illness conversation with their oncologist. We found no evidence of harm and found that serious illness conversations were considered standard of care for patients with cancer.

Verbal consent will be obtained from each clinician who participates in the semi-structured interviews. See **Conversation Connect Initiative Clinician Interview Guide** for the template for verbal consent. Written consent is not necessary for this project because clinicians will not be required to participate and can opt out at any point. No patient identifying information will be provided to clinicians.

8.2 Procedures

Data on oncologists and their patients at the University of Pennsylvania Health System will be obtained from Penn Data Store and Clarity (Epic's data reporting database). Physician data includes demographic information (e.g. sex, type of medical degree, etc.) and may be also obtained from publicly available databases or websites online. The predictive algorithm identifies high-risk patients based on demographic information, information about comorbid conditions (including type of cancer; other variables like diabetes, hypertension, and chronic kidney disease, and comorbid conditions needed to calculate the Charleston Comorbidity Index; laboratory test results; and previous emergency department and hospital admissions. This

predictive algorithm has been validated and results are currently being submitted for publication in a medical journal. Clarity will be used to identify documentation of SICs and ACP.

After identifying eligible oncologists, block randomization will occur at the clinic level (noting that PCAM melanoma and CNS Oncology will be randomized together as both clinics have a low number of providers). We will obtain baseline measures and plan to stratify the randomization by those above and below median level of SICPs in March through May of 2019.

9. Analysis plan

All analyses will be conducted using intention-to-treat using the patient as the unit of analysis and clustering at the level of the oncologist. Advanced practice providers (APPs) will receive the intervention, but will be associated with the oncologist with whom they work for the purposes of the analysis. All hypothesis tests will use a two-sided alpha of 0.05 as our threshold for statistical significance.

The primary and secondary outcome measures will use a binary indicator representing the presences of an SIC or ACP for each patient. The primary outcome will be expressed as a standardized rate of documented SIC discussions (number of documented SIC notes / 100 unique patient visits). In the main adjusted analysis, we will fit models using generalized estimating equations cluster on oncologists, using group (oncology practices) and period (4-week increments) fixed effects and adjusting for monthly temporal trends.

To test the robustness of our findings, we will perform sensitivity analyses that adjusts for available patient characteristics and comorbidities such as demographics and the Charlson Comorbidity Index.

Additional sensitivity analyses will include:

- Including patients enrolled in aforementioned palliative care lung cancer trial
- Analyzing results clustering at the level of the clinician (oncologist or APP)

We will use descriptive statistics to analyze responses from our REDCap surveys.

The semi-structured interviews will be transcribed and uploaded to NVivo12 Plus, a data management software. We will use a modified content analysis approach with constant comparative coding to analyze the interview transcripts. We will iteratively develop a codebook following the initial interviews based on the structure of the interview guide. One of two reviewers will code each transcript, and approximately 20-25% of total transcripts will be coded by both reviewers to establish inter-reviewer reliability. The adequacy of the codebook will be periodically assessed by the reviewers in partnership with the research team and modifications will be made as their understanding of the data and emergent themes evolves over time. The reviewers will meet regularly to discuss discrepancies and update the code book as needed, with record keeping adequate to track changes to the code book and the rationale. Kappa statistics will

be generated to estimate inter-rater reliability. Coding will then be reviewed to summarize key themes, and representative quotes will be selected to illustrate those themes.

To assess the impact of the COVID-19 pandemic on rates of conversations, we will use a quasi-experimental interrupted time series analysis, using March 23rd, 2020 as the date of the COVID exposure, to assess whether the decline in overall risk scores and number of patients flagged as high risk is significantly lower during the COVID pandemic. We will use descriptive statistics to compare the distribution of demographic characteristics of high-risk patients (Conversation Connect score >10%) before and after the pandemic. We will use an interrupted time series analysis with historic control to assess the impact of the COVID pandemic on the accuracy of the Conversation Connect model; we will use several metrics (AUC, PPV, sensitivity) to define accuracy. Finally, we will use an interrupted time series analysis with historic control to assess the impact of the COVID pandemic and associated changes in risk scores with rates of serious illness conversations documented.

10. Investigators

Ravi Parikh, MD, MPP is the Principal Investigator. Dr. Parikh is an Assistant Professor in the Department of Medical Ethics and Health Policy and Medicine at the University of Pennsylvania with experience implementing pragmatic clinical trials of similar scale at the University of Pennsylvania Health System. Christopher Manz, MD, MSHP and Mitesh Patel, MD, MBA, are the co-Investigators. All investigators have experience implementing similar pilot interventions as quality improvement initiatives at Chester County Hospital and Penn-Presbyterian Medical Center in 2018.

Dr. Manz and Dr. Parikh are supported by the Conversation Connect team and Abraham Cancer Center leadership, including:

Nina R. O'Connor, MD	Palliative Care
Justin E. Bekelman, MD	Penn Center for Cancer Care Innovation
Michael Draugelis, MS	Penn Data Science
Mitesh Patel, MD, MBA	Penn Nudge Unit
Lynn M. Schuchter, MD	Hematology/Oncology
Lawrence N. Shulman, MD	Hematology/Oncology
Sujatha Changolkar	Penn Nudge Unit
Corey Chivers, PhD	Penn Data Science
Susan Harkness Regli, PhD	Human Factors
Cody Cotner	Perelman School of Medicine
Lead Biostatistician (TBD)	

11. Human research protection

11.1 Data confidentiality

Computer-based files will only be made available to personnel involved in the study through the use of access privileges and passwords. Wherever feasible, identifiers will be removed from study-related information. Precautions are already in place to ensure the data are secure by using passwords and HIPAA-compliant encryption.

11.2 Subject confidentiality

Data on physicians and patients will be obtained from Epic, Penn Data Store and Tableau. Any information that is obtained will be used only for research purposes and to inform the behavioral nudges described above. Information on individual patients will only be disclosed within the study team. All study staff will be reminded of the confidential nature of the data collected and contained in these databases.

Data regarding provider performance of Serious Illness Conversations are already shared among providers and will continue to be shared in unblinded fashion as part of the trial. Data regarding acute care utilization in the last 30 days for a provider's deceased patient panel will be shared amongst providers as well. This will occur as part of the intervention but is planned to occur occur regardless of trial approval as part of quality improvement efforts.

Data will be stored, managed, and analyzed on a secure, encrypted server behind the University of Pennsylvania Health System (UPHS) firewall. The primary investigator (Dr. Patel) and statistical analyst will be blinded to the randomization schema and which groups are receiving the intervention. This server was created for projects conducted by the Penn Medicine Nudge Unit related to physician and patient behavior at UPHS. All study personnel that will use this data are listed on the IRB application and have completed training in HIPAA standards and the CITI human subjects research. Data access will be password protected. Whenever possible, data will be deidentified for analysis.

11.3 Subject privacy

All efforts will be made by study staff to ensure subject privacy. Data will be evaluated in a de-identified manner whenever possible. We will require time and date of appointment and zip code data of trial participants to define our exposure period and link to area-level socioeconomic data from the American Community Survey.

11.4 Data disclosure

Information on physicians and patients will not be disclosed to anyone outside of the study team, with the exception of provider level data (SIC rates, acute care utilization) that are deliberately shared as a part of the behavioral nudges.

11.5 Data safety and monitoring

The investigators will provide oversight for the study evaluation of this health system initiative. Providers will use their clinical judgment to determine the appropriateness of initiating ACPs with patients, in accordance with standard of care.

11.6 Risk/benefit

11.6.1 Potential study risks

The potential risks associated with this study are minimal. Breach of data is a potential risk that will be mitigated by using HIPAA compliant and secure data platforms for the nudge interventions (name of list platform and platform used to share info w/ MAs) and evaluation (Nudge Unit server). As noted above, substantial data demonstrates that ACPs improve patient goal-concordant care without any identified harms (despite concerns that ACPs may increase psychosocial distress, the opposite has been found), so the negative impact on patients is minimal.

The provider data that will be shared with providers is already shared in one form (in the case of SIC rates) and is planned to be shared with providers in the near future independent of this trial (in the case of acute care utilization near the end of life), so the trial does not exposure providers to additional risk.

11.6.2 Potential study benefits

As described in the literature, patients may have improved quality of life and better goal-concordant care when exposed to ACPs, especially earlier in their disease course. An intervention that prompts providers to have an ACP with patients at a high risk of death in the next six months may increase the likelihood that these conversations occur and that they occur earlier in the disease course. However, it is possible that patients will receive no benefit from this study.

11.6.3 Risk/benefit assessment

The risk/benefit ratio is highly favorable given the potential benefit from eligible patients having an SIC or ACP discussion with their provider and benefitting from better goal-concordant care and that efforts have been put into place to minimize the risk of breach of data.
