# Supplementary material for: Long-term Effect of Machine Learning–Triggered Behavioral Nudges on Serious Illness Conversations and End-of-Life Outcomes Among Patients With Cancer: A Randomized Clinical Trial
Source: JAMA Oncol. 2023 Jan 12;9(3):414–8. doi: 10.1001/jamaoncol.2022.6303 (PMC9857721; doi:10.1001/jamaoncol.2022.6303)
Supplement: Supplement 2. — eTable 1. Characteristics of Patients With Clinician Encounters eTable 2. Subgroup Analyses [file jamaoncol-e226303-s002.pdf]

Supplemental Online Content

Manz CR, Zhang Y, Chen K, et al. Long-term effect of machine learning–triggered behavioral nudges on serious illness conversations and end-of-life outcomes among patients with cancer: a randomized clinical trial. *JAMA Oncol.* Published online January 12, 2023. doi:10.1001/jamaoncol.2022.6303

eTable 1. Characteristics of Patients With Clinician Encounters

eTable 2. Subgroup Analyses

This supplemental material has been provided by the authors to give readers additional information about their work.

© 2023 Manz CR et al. *JAMA Oncology.*

eTable 1. Characteristics of Patients With Clinician Encounters

Characteristic	All patient encounters, No. (%)		Standardized mean difference	Encounters with high-risk patients, No. (%)[a]		Standardized mean difference	Encounters with non–high-risk patients, No. (%)		Standardized mean difference
	Control (n = 12 356)	Intervention (n = 28 665)		Control (n = 1754)	Intervention (n = 3766)		Control (n = 10 602)	Intervention (n = 24 899)	
Age, mean (SD), y	62.5 (13.9)	61.6 (14.5)	−0.06	65.4 (12.7)	64.4 (13.2)	−0.08	62.0 (14.0)	61.1 (14.7)	−0.05
Female	6515 (52.7)	15 744 (54.9)	0.04	865 (49.3)	1875 (49.8)	0.01	5650 (53.3)	13 869 (55.7)	0.04
Male	5841 (47.3)	12 921 (45.1)		889 (50.7)	1891 (50.2)		4952 (46.7)	11 030 (44.3)	
Race and ethnicity			0.03			0.04			0.03
Non-Hispanic Black	2496 (20.2)	5679 (19.8)		381 (21.7)	801 (21.3)		2115 (19.9)	4878 (19.6)	
Non-Hispanic White	8765 (70.9)	20 142 (70.3)		1239 (70.7)	2674 (71.0)		7526 (71.0)	17 468 (70.2)	
Other[b]	1095 (8.9)	2844 (9.9)		134 (7.6)	291 (7.7)		961 (9.1)	2553 (10.3)	
Insurance			0.08			0.08			−0.02
Commercial	5447 (44.1)	13 383 (46.7)		634 (36.1)	1469 (39)		4813 (45.4)	11 914 (47.8)	
Medicare	6157 (49.8)	13 324 (46.5)		1007 (57.5)	2010 (53.3)		5150 (48.6)	11 314 (45.4)	
Medicaid	752 (6.1)	1958 (6.8)		113 (6.4)	287 (7.7)		639 (6.0)	1671 (6.7)	
Cancer type[c]			0.69						0.76
Breast	2034 (16.5)	3657 (12.8)		252 (14.3)	399 (10.6)	0.73	1782 (17.0)	3258 (13.0)	
Central nervous system + melanoma	778 (6.3)	2268 (7.9)		98 (5.6)	281 (7.5)		680 (6.4)	1987 (8.0)	
Gastrointestinal	660 (5.3)	4558 (15.9)		119 (6.7)	828 (21.9)		541 (5.1)	3730 (15.0)	
General oncology	2203 (17.8)	7297 (25.5)		223 (12.6)	650 (17.3)		1980 (19.0)	6647 (27.0)	
Genitourinary	1829 (14.8)	1803 (6.3)		265 (15.3)	226 (6)		1564 (15.0)	1577 (6.3)	
Myeloma	2609 (21.1)	2459 (8.6)		403 (23.1)	389 (10.3)		2206 (21.0)	2070 (8.3)	
Lymphoma	542 (4.4)	4065 (14.2)		60 (3.4)	424 (11.3)		482 (4.5)	3641 (15.0)	
Thoracic	1701 (13.8)	2558 (8.9)		334 (19)	569 (15.1)		1367 (13)	1989 (8.0)	

© 2023 Manz CR et al. *JAMA Oncology.*

Charlson Comorbidity Index score, median (IQR)	3 (2-8)	2 (2-8)	−0.11	8 (3-9)	8 (3-9)	−0.15	2 (2-8)	2 (2-5)	−0.09
Predicted mortality risk, median (IQR)[d]	3.5 (1.6-11.2)	2.5 (1.4-8.4)	−0.06	30.0 (19.1-46.7)	28.0 (16.0-46.0)	−0.07	3.0 (1.0-6.0)	2.0 (1.0-5.0)	−0.12
Encounters with high-risk patients (% of total)	1754 (14.2)	3766 (13.1)	NA	NA	NA	NA	NA	NA	NA

Abbreviation: NA, not applicable.

[a]High-risk patients were defined as having a 10% or greater risk of death within 180 days, as identified by a prospectively validated machine learning algorithm.

[b]Other included American Indian, Asian, East Indian, Hispanic Black, Hispanic White, and Pacific Islander.

[c]Imbalances between control and intervention groups were due to staggered intervention start times in the stepped-wedge design.

[d]Mortality risk at 180 days in absolute percentage points, as predicted by the mortality prediction algorithm.

© 2023 Manz CR et al. *JAMA Oncology.*

eTable 2. Subgroup Analyses

Serious Illness Conversation	Control	Intervention	Adjusted Odds Ratio for Intervention Relative to Control (95% CI)[a]	P Value[b]
**All patient encounters**	1.3% (158/12356)	4.4% (1262/28665)	2.09 (1.53-2.87)	<.0001
**Fully Adjusted Model**	-	-	2.09 (1.53-2.87)	<.0001
**Subgroup analyses**				
Age				
18-35 years	0.4% (3/702)	1.7% (34/2010)	Ref	Ref
36-50 years	1% (16/1568)	2.7% (111/4109)	0.68 (0.18-2.54)	0.5621
51-64 years	1.5% (64/4186)	4.6% (428/9301)	0.79 (0.24-2.67)	0.7102
≥ 65 years	1.3% (75/5900)	5.2% (689/13242)	1.12 (0.35-3.6)	0.855
Gender				
Female	1.3% (86/6515)	4% (625/15744)	Ref	Ref
Male	1.2% (72/5841)	4.9% (637/12921)	1.28 (0.84-1.96)	0.2476
Race/Ethnicity				
White non-Hispanic	1.3% (111/8765)	4.2% (856/20142)	Ref	Ref
Black non-Hispanic	1.5% (37/2496)	4.7% (267/5679)	0.93 (0.54-1.61)	0.7936
Other	0.9% (10/1095)	4.9% (139/2844)	1.55 (0.59-4.05)	0.3722
Insurance				
Commercial	1.2% (67/5447)	3.4% (456/13383)	Ref	Ref
Medicare	1.4% (84/6157)	5.3% (705/13324)	1.5 (0.96-2.35)	0.0732
Medicaid	0.9% (7/752)	5.2% (101/1958)	2.19 (1.1-4.37)	0.0265
Predicted mortality risk				
0.0 - 0.1	0.4% (32/8413)	1.3% (276/20865)	Ref	Ref
>0.1 to 0.2	1.6% (23/1417)	8% (216/2699)	1.67 (0.94-2.96)	0.0784
>0.2 to 0.3	1.7% (11/647)	14% (162/1154)	2.81 (1.45-5.45)	0.0022
>0.3 to 0.4	3.9% (15/386)	17.3% (129/745)	1.64 (0.82-3.3)	0.1624
>0.4 to 0.5	4.7% (12/253)	20.9% (109/522)	1.79 (0.67-4.78)	0.2434
>0.5	12% (47/391)	32.4% (275/850)	1.06 (0.56-2.01)	0.8582
Marital Status				

© 2023 Manz CR et al. *JAMA Oncology.*

Single	1.7% (42/2457)	4% (245/6056)	Ref	Ref
Married	1.1% (86/7753)	4.2% (737/17699)	1.59 (0.98-2.57)	0.0611
Other	1.4% (30/2146)	5.7% (280/4910)	1.76 (0.87-3.54)	0.1153
**High-risk patient encounters**	3.4% (59/1754)	13.5% (510/3765)	2.62 (1.84-3.72)	<.0001
**Fully Adjusted Model**	-	-	2.62 (1.84-3.72)	<.0001
**Subgroup analyses**				
Age[c]				
18-35 years	0% (0/50)	15.3% (21/137)		
36-50 years	0.8% (1/131)	11.6% (39/335)		
51-64 years	5.7% (32/558)	11.9% (142/1191)		
≥ 65 years	2.6% (26/1015)	14.7% (308/2102)		
Gender				
Female	4% (35/865)	13.9% (260/1869)	Ref	Ref
Male	2.7% (24/889)	13.2% (250/1896)	1.41 (0.66-3.02)	0.3776
Race/Ethnicity				
White non-Hispanic	3.6% (45/1240)	12.8% (341/2662)	Ref	Ref
Black non-Hispanic	3.2% (12/380)	16.1% (130/809)	1.44 (0.49-4.19)	0.5067
Other	1.5% (2/134)	13.3% (39/294)	2.43 (0.32-18.2)	0.3889
Insurance[c]				
Commercial	4.4% (28/633)	10.7% (157/1468)		
Medicare	3.1% (31/1008)	14.8% (297/2007)		
Medicaid	0% (0/113)	19.3% (56/290)		
Marital Status				
Single	4.6% (16/346)	14.8% (108/730)	Ref	Ref
Married	3.5% (38/1076)	12.8% (289/2261)	1.18 (0.47-2.96)	0.7192
Other	1.5% (5/332)	14.6% (113/774)	3.2 (0.96-10.64)	0.0576

[a] Subgroup analyses adjusted for intervention, clinic group, and wedge, and clustered at the oncologist level.

[b] P-values are from interaction models, adjusted for intervention, clinic group, wedge, and subgroup:intervention interaction term.

[c] Odds ratios and p-values could not be generated because regression models could not generate results if subgroups had low sample size

© 2023 Manz CR et al. *JAMA Oncology.*
