# Supplementary material for: Long-term Effect of Machine Learning–Triggered Behavioral Nudges on Serious Illness Conversations and End-of-Life Outcomes Among Patients With Cancer: A Randomized Clinical Trial
Source: JAMA Oncol. 2023 Jan 12;9(3):414–8. doi: 10.1001/jamaoncol.2022.6303 (PMC9857721; doi:10.1001/jamaoncol.2022.6303)
Supplement: Supplement 3. — Data Sharing Statement [file jamaoncol-e226303-s003.pdf]

Data Sharing Statement

Manz. Long-term Effect of Machine Learning-Triggered Behavioral Nudges on Serious Illness Conversations and End-of-Life Outcomes Among Patients With Cancer. *JAMA Oncol*. Published January 12, 2023. doi:10.1001/jamaoncol.2022.6303

Data
Data available: Yes
Data types: Deidentified participant data
How to access data: ravi.parikh@pennmedicine.upenn.edu
When available: With publication

Supporting Documents
Document types: None

Additional Information
Who can access the data: researchers whose proposed use of the data has been approved
Types of analyses: Secondary data analysis
Mechanisms of data availability: After approval of a proposal and with a signed data access agreement
Any additional restrictions: None
